# Sarcoptic-mange detector dogs used to identify infected animals during outbreaks in wildlife

**DOI:** 10.1186/1746-6148-8-110

**Published:** 2012-07-09

**Authors:** Samer Alasaad, Roberto Permunian, Francis Gakuya, Matthew Mutinda, Ramón C Soriguer, Luca Rossi

**Affiliations:** 1 Institute of Evolutionary Biology and Environmental Studies (IEU), University of Zürich, Winterthurerstrasse 190, 8057, Zürich, Switzerland; 2 Dipartimento di Produzioni Animali, Epidemiologia ed Ecologia, Università degli Studi di Torino, Via Leonardo da Vinci 44, I-10095, Grugliasco, Italy; 3 Department of Veterinary and Capture Services, Kenya Wildlife Service, Nairobi, Kenya; 4 Estación Biológica de Doñana, Consejo Superior de Investigaciones Científicas (CSIC), Avda. Américo Vespucio s/n 41092, Sevilla, Spain

**Keywords:** *Canis lupus* var. *familiaris*, *Sarcoptes scabiei*, *Rupicapra pyrenaica*, Forensic science, Disease surveillance, Animal conservation

## Abstract

**Background:**

One of the main aims of forensic investigation is the detection and location of people and substances of interest, such as missing people and illegal drugs. Dogs (*Canis lupus* var. *familiaris*) have had an important role in legal and forensic investigations for decades; nonetheless canines’ keen sense of smell has never been utilized in either the surveillance or control of wildlife diseases. The rapid removal and treatment of infected carcasses and/or sick animals is a key task in the management of infectious diseases, but it is usually difficult or impractical to carry out in the wild.

**Results:**

In this paper we report on a study running over a period of 15 years, in which - for the first time to our knowledge - two disease-detector dogs were trained to follow the scent of *Sarcoptes*-infected animals and to find carcasses, even under the snow, and apparently no false positives were detected in fieldwork. Sarcoptic mange-detector dogs were used to collect the carcasses of 292 mangy wild animals and to identify, separate from their herd, and capture 63 mange-infected wild animals in the Italian Alps.

**Conclusions:**

Properly trained disease-detector dogs are an efficient and straightforward tool for surveillance and control of sarcoptic mange in affected wild animal populations.

## Background

Despite the fact that the first known scientific experiment using dogs’ olfactory abilities dates back to the late nineteenth century [1], only limited scientific research has ever taken advantage of these animals’ sense of smell (e.g. [2,3]). Air-scent detection dogs are widely used by law enforcement agencies to identify narcotics, explosives, and contraband, and also by fire investigators to detect the presence of accelerant materials. Detector canines are also used by police, military, and rescue service to locate missing or lost peoples, natural or mass disasters victims, and for locating partial scattered human remains [4]. Thirty kinds of detector dogs have been documented [2], and recently dogs were used for bed bugs detection as a safer alternative to blind pesticide use [5], but to the best of our knowledge there are no cases of dogs having been trained to detect wildlife diseases.

In the field of wildlife conservation, there are two key factors in disease monitoring and control (when the latter is feasible or desirable): (i) the rapid detection and removal of infected carcasses, and (ii) the rapid and accurate identification, separation and capture of the infected animals for treatment or euthanasia, if indicated [6-8]. Both infected carcasses and sick animals are potential sources of infection for conspecifics, the other sympatric animals and even humans [9-11]. The fore mentioned mandatory tasks are often difficult and/or impracticable to accomplish, especially: (a) when carcasses are scattered across a wide geographic area and are difficult to spot: e.g. due to presence of snow-cover or thick vegetation, (b) in cases involving shy and social species, : e.g. in which attempts to segregate sick animals from the rapidly escaping herds they belong to, are usually unsuccessful and, if repeated, may also favor spreading of the disease due to disturbance [12]. And (c) when the diseases in question have no visible external symptoms: e.g. in the case of animals where initial skin lesions are occulted by a long fur [13].

The ubiquitous ectoparasite *Sarcoptes scabiei* infect more than 100 species of mammals, worldwide [14,15]. It is a neglected emerging and re-emerging parasite [16,17], threatening the conservation of global biodiversity [18]. In *Sarcoptes*, host specificity is a long lasting matter of debate [9,19-22], and epidemiological patterns clearly differ from one area or animal species to another [14,23]. Amongst consequences of *Sarcoptes* uncontrolled spreading are severe mortality in wild and (poorly managed) domestic animals [12,24].

*Sarcoptes* mite infections are endemic in many wild animals, above all in canids in North America, Europe and Australia, cats in Europe and Africa, ruminants and wild boars in Europe, ruminants and great apes in Africa, wombats and koalas in Australia [12]. *Sarcoptes* infection in these hosts is accompanied, amongst other signs, with extensive hyperkeratosis and the formation of thick crusts. Lesions easily get infected with bacteria and animals develop a foul aromatic odour [13,14]. For parasitologists, the odour of a mangy animal is unique and distinguishable (even no specific study has been carried out in this direction), hence it seemed reasonable to figure out that dogs could be trained to recognize and localize the sources of such special odour under field conditions.

The aim of this paper is to report the use of trained disease-detector dogs in a sarcoptic mange (scabies) outbreak area in the Alps, where they significantly contributed to enhance the level of disease surveillance and control compared with traditionally available tools. Our study was empirically based and originated from an immediate need of the local wildlife service at the beginning of an unexpected sarcoptic mange outbreak.

## Methods

### Study area

In 1995, Northern chamois (*Rupicapra rupicapra*) living in the Dolomite Alps, Italy, were naturally exposed for the first time to a sarcoptic mange outbreak (by *S. scabiei* var. *rupicaprae*). This study was conducted in a remote portion of the municipality of Auronzo di Cadore, in the province of Belluno (13,500 hectares, 46°33’0”N 12°26’0”E), where the index case of the outbreak was found. Approximately 85% of the area is above 1,000 m a.s.l. (range 600–3,300 m a.s.l.) and 45% is covered by forests. Mean annual temperature ranges between 6 and 8 °C at 1,000–1,200 m a.s.l., whereas mean annual rainfall ranges from 1,025 to 1,400 mm, with relatively dry winters and most of the precipitation occurring during summer and early autumn. Presence of snow usually exceeds 150 days at 1,000 m a.s.l.

**Figure 1:**
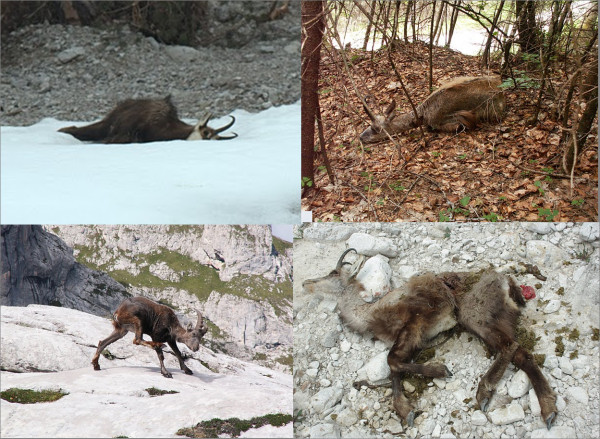
Photos of decomposed carcasses, and sick Alpine chamois and Alpine ibex showing mangy lesions.

At that time of the index case, conservative census data by direct observation indicated that the local chamois population, for which yearly census data by direct observation were available, was numbering 810 heads (6.0 chamois/100 ha). In the following years, a significant demographic decline (with peak mortality in years in 1997, 1998 and 2001) was observed, leading to a low post-epidemic density of 1.4 heads/100 ha and a conservative decline estimate of 77% (Figure 1). The first epidemic wave of scabies waned during years 2003 to 2007, but a second wave was observed in years 2008 to 2010. Mortality of chamois during the second wave was considerably lower, and a 25% decline (from 4.8 to 3.6 heads/100 ha) was estimated. *Sarcoptes* infection was also recorded in the Alpine ibex (*Capra ibex*) and, sporadically, in other sympatric wild ruminants (*Cervus elaphusCapreolus capreolus* and *Ovis aries musimon*). Scabies is still persisting in the area [25].

### Dog training

We trained two Bayerischer Gebirgsschweisshund (Bavarian_Mountain_Hound) dogs to perform two tasks: (a) locate carcasses of mangy chamois and (b) identify and eventually separate (from their herds), and capture the highly contagious sick animals. This breed was chosen since these dogs are known to be strong, agile and tolerant of the cold mountain weather.

Before the onset of mange in the Dolomites, Ingo (four-year-old male dog) had been trained and used to search and locate wounded ungulates during the hunting season, above all chamois and roe deer. At least once a week, Ingo was trained to work on “true” artificial trail over 300 m in length at the end of which an ungulate carcass (usually a red deer, *C. elaphus*) was placed, as well as on “false” trail without any final carcass. The dog was accustomed to operate with a "belt" (a 10-m-long leash) for up to two-thirds the length of the trail (~ 200 m), and was then unleashed. Ingo had been trained to bark in the proximity of the carcass, which prompted the giving of a reward, food and play (Pavlovian conditioning).

**Figure 2:**
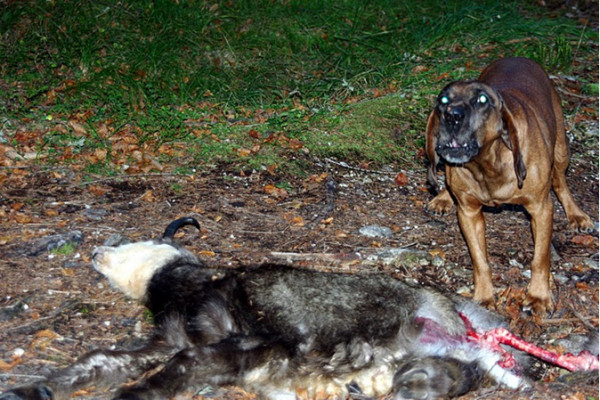
Photo of laboratory-like conditions, showing the trained dogs with the carcasses of mangy Alpine chamois.

The same training scheme, with the only deviation that mangy carcasses were used for “true” trails and mange-free carcasses for “false” trails, was used to accustom Ingo to the exclusive search of infected animals. At first, Ingo barked whenever he found a carcass (mangy or mangy-free), but he was only rewarded when successful in localizing a mangy carcass. Additional training was carried out with mangy carcasses only. A mange-infected carcass was located in a known place, ~300 m far from the dog (in some cases under snow or in the dense forest). The dog was trained to search and locate the mangy carcass, and he was taught to approach to mangy carcass’s pulling away point (the nearest point to pull away the carcass from the snow). By the end of the specific training phase, Ingo had learned to seek out only the mangy carcasses, even under the snow cover. His ability to track and stop the living mangy chamois was similar to his original work to recover wounded animals in a hunt context, but in this case mangy animals were the “targets” and healthy ones the “false” (Figure 2). Ingo had 3–4 training sessions each week for approximately 6 months. The training session took between 5 minutes, when carcasses were near to the street or they were approximately localized by forest rangers and mountaineers, and up to 8 hours in the isolated localities. Finally, the instinct to detect mange-infected animals was strengthened by voluntarily bringing Ingo into contact with sick living chamois, and practicing the euthanasia (humane shooting) in his presence. Ingo has learned to recognize severely mangy animals as "easy prey", and his skills at searching for this category became well-honed. Overall, training of Ingo took approximately three months.

Another male Bayerischer Gebirgsschweisshund (Buck, three-month-old, he is now 12 year-old), joined Ingo (who died in 2001) in summer 1999, in the mission of detecting mange-infected animals. Buck was trained in the same way as Ingo. Work-shadowing and “learning by contiguity” enabled this puppy the chance to become disease-detector dog much faster than Ingo. Buck had 2–3 real training sections daily for 3 months, coinciding with the peak of *Sarcoptes* epidemic wave in the study area. When Buck was five months old, he detected his first mangy carcass under packed snow (4 November 1999).

### Field-work

We used two techniques for locating and collecting mangy carcasses and for identifying, separating and eventually capturing the mangy animals for sampling or euthanasia purposes:

(i) Handler-dog system (for both carcasses and live mangy animals): Handler and dog walked together along forest paths, mainly following the valley bottoms. Paths were generally walked from the highest to the lowest altitude, to facilitate dogs in picking up the scents carried by the wind. On finding a mangy animal, which is still alive, the dogs follow down to a distance of 10–25 m to permit proper approach by the handler. Moreover, the dogs were trained to move in a zigzag fashion, so that actually walked area was expanded to a width of 50–100 m.

(ii) Free-dog system (only for live mangy animals): Dogs were left to work freely, since mangy chamois seem to have less fear of dogs than humans and the presence of a handler would probably scare the animals. Moreover, freely ranging disease-detector dogs barked when faced with a live mangy animal, thus allowing the handler to identify the site and ease capture of the animal.

The handler-dog system was used to localize the carcasses of the dead animals affected with *Sarcoptes*, while the free-dog system was used to identify and separate the mangy animals from their herd.

### Parasite collection and disease confirmation

Affected areas of skin from the collected animals were scraped with scalpel crusts for parasitological examination. The scrapings were placed in universal bottles containing 70% ethanol and transported to the laboratory. A portion was removed from the alcohol and subjected to KOH to recover parasites for microscopy [26]. The collected mites were identified as *S. scabiei* on the basis of known morphological criteria [27].

## Results

Sarcoptic mange-detector dogs were used to identify, separate from their herd and selectively euthanize 63 mangy chamois, and to collect the carcasses of further 292 mangy individuals in the Italian Alps (Figure 4). In the same period, local hunters selectively culled only 18 mangy chamois, and further 65 mangy carcasses were found in the frame of passive and active surveillance conducted with “traditional” methods (namely without dogs). Apparently no false positives were detected in fieldwork. Dogs were able to localize chamois even when mildly affected (eg, in cases in which the true cause of the death was winter starvation, and only patches of scabietic skin were present).

**Figure 3:**
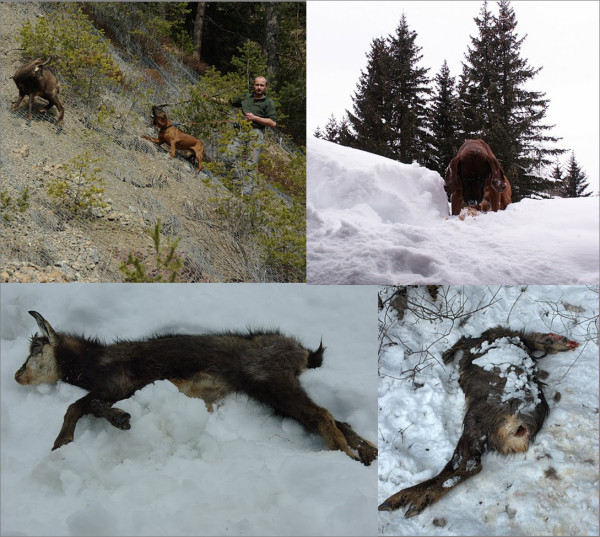
**Photos showing the handler and the trained disease-detector dogs in the field, and a number of Alpine chamois carcasses collected from under the snow cover.** The handler, Roberto Permunian, is consented to the use of his image for publication purposes.

The reliability of infected animal identification by disease-detector dogs (the diagnostic ratio) is the percentage of the correct identification of mangy carcasses or live animals [28]. Although no statistical analysis can be performed regarding the reliability of *Sarcoptes* scent identification by our dogs, we can report that in no case did the *Sarcoptes*-detector dogs misdiagnose mange infection. All carcasses and live animals identified by the dogs as mangy were confirmed as having sarcoptic mange lesions upon post-mortem examination [27].

On several occasions, forest rangers and mountaineers alerted us to the presence of a sick animal at a certain location. Our dogs were extremely performant in identifying and follow the track of sick animals (even many hours from after the alarm had been raised), and then separate them to allow euthanasia (Figure 3).

**Figure 4:**
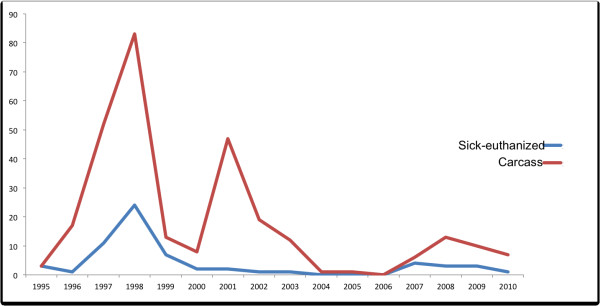
Number of mangy carcasses and euthanized Alpine chamois identified, separated from the herd, and captured with the aid of disease-detector dogs between 1995 and 2010.

**Figure 5:**
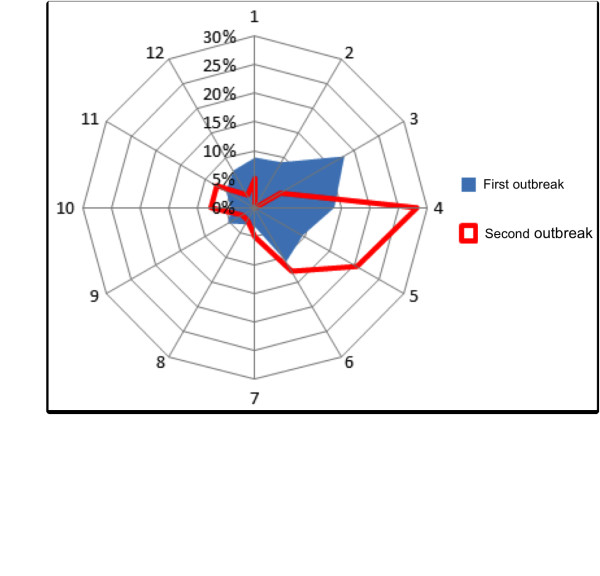
**Monthly patterns of mangy carcasses and euthanized Alpine chamois localized with the aid of disease-detector dogs between 1995 and 2010.** Blue color represents the first *Sarcoptes* epidemic wave between 1995 and 2000. Red color represents the second epidemic wave between 2000 and 2010.

The patterns of detection of carcasses and mangy animals varied over time. The recovery rate varied between the years of the study, with two peaks in 1997 and 2001 (for more details see Figure 4). More carcasses were collected in spring (mainly March and April, 36.6%) and relatively fewer in summer (18.3%), fall (15.3%) and winter (18.3, 15.3 and 20.6%, respectively). Mange-infected animals (to be euthanized) were found mostly in winter and spring (72.4%) (For more details see Figure 5). Disease-detector dogs identified more mangy females than males, with a sex bias of 1:0.71 female–male.

In different districts of the Dolomites, ≥68.5% (±12.6 sd, range 55.0-93.5%) are usually lost to surveillance [25], but this proportion dropped to an estimated ≥38.9% in the study area, which was the single district where dog-aided personnel was dedicated to this task.

## Discussion

Despite technological advances, dogs’ remarkable olfactory abilities are still used by a wide range of investigators and detectors [2]. Statistical testing of this ability shows that dogs are capable of matching scents under very different circumstances (e.g. [29,30]).

Properly trained disease detector-dogs were used faultlessly to locate and/or collect the carcasses of 292 mangy wild animals, and to identify, separate (from their herd), and capture 63 *Sarcoptes*-infected wild animals in a remote part of the Italian Alps. Though the recovery rate of carcasses which were available in the field could not be precisely defined, estimates of the carcasses which were lost to surveillance during this scabies outbreak, based on all type of recovery and the pre- and post-epidemic number of chamois, indicated that the use of dogs substantially enhanced recovery rates. Obviously, the handler’s experience and dedicated time, and the heath of the dogs may play a crucial role in the detection rate of mangy animals.

The patterns of detection of carcasses and mangy animals varied over time, with two peaks in 1997 and 2001, coincident with the peaks of *Sarcoptes* epidemic waves in the affected wild population [25]. Disease-detector dogs identified more mangy females than males. In fact, the basis of the sex ratio was not a sex-biased difference in sensitivity to scabies or a greater accuracy of dogs in detecting females. Simply, this was the result of a female-biased sex ratio in the affected chamois population, as usual in the majority of districts were hunting is allowed. More carcasses were collected in spring and relatively fewer in summer, fall and winter, and this again related to the higher prevalences of affected chamois in spring compared with the other seasons [25]. Interestingly, the regular use of dogs showed that occurrence of mange is not as concentrated in winter and early spring as observations from distance to detect scabietic chamois would suggest [25].

Under the circumstances of this study, in which a deadly wave of scabies affected a naive and dense chamois population, a large number of carcasses and live mangy animals was available, beyond other purposes, also for dog training. This allowed continuous reinforce of the elements necessary for olfactory discrimination. However, should “fresh” material be infrequently available for dog training, thawing frozen mangy carcasses may represent a valuable alternative, as per experience in this study [3,31].

It was vital to reward the dogs by allowing them to get in contact with the located mangy carcasses and captured mange-infected animals. Despite potential risk of developing annoying pseudo-mange, just as it occurred in involved personnel [11], dogs in this study did never show any compatible signs.

Though efficacy of early selective culling of mangy chamois is questionable, this measure is recommended in the management of several hunting estates across the Alps [32]. Unfortunately, selective culling implies a reliable diagnosis from distance, and this is often hampered by “occult” presentations of the disease (e.g., crusty lesions embedded in thick fur or localized in poorly visible region, as the abdomen and inguine) generating false negatives [33]. As shown in this study, properly trained *Sarcoptes*-detector dogs are of great support in the detection of these early presentations of mange, and in the action leading to selective removal of affected chamois.

## Conclusions

Twelve thousand years after the first use of dogs for hunting [2], we report here for the first time to our knowledge, the ability of disease-detector dogs to detect/locate mange-infected carcasses, and identify, separate and capture mangy animals. As anticipated, in this study the training and use of disease detector-dogs was dictated by an emergency and stimulated by environmental difficulties typical of the high mountains, hence further studies are needed to precisely identify the sensibility and specificity of such method.

In summary, disease-detector dogs are a potentially useful tool in wildlife disease surveillance and control. They are able to track animals over a wide geographic area in rugged terrain and can detect diseased animals that are not showing overt clinical symptoms. We expect further ramifications of disease-detector dogs in a wider range of animal and human disease scenarios.

## Ethical approval

Mangy animals capturing and euthanizing was under the National Law for Hunting in Italy (Law N. 157), and the Regional Law for Hunting in Regione Veneto (Law N. 50).

## Competing interests

The authors declare that they have no competing interests.

## Authors’ contributions

RP and LR conceived and designed the experiments. RP, LR, SA, FG, MM and RC performed the fieldwork experiments. Manuscript was analysed, discussed and written by all co-authors. All authors read and approved the final manuscript.
